# Differential Thigmotropic Capacities Among Fusarial Isolates: *Fusarium solani* Species Complex (FSSC) Isolates Most Potent

**DOI:** 10.3390/jof12050344

**Published:** 2026-05-07

**Authors:** Sehee I. Rim, Matthew L. Duley, Michael R. Hughes, Jing Jing, Marcia R. Lee

**Affiliations:** 1 Department of Microbiology, Miami University, Oxford, OH 45056, USA; srim.dds@gmail.com; 2 Center for Advanced Microscopy & Imaging, Miami University, Oxford, OH 45056, USA; duleyml@miamioh.edu; 3 Statistical Consulting Center, Miami University, Oxford, OH 45056, USA; hughesmr@miamioh.edu (M.R.H.); jingj4@miamioh.edu (J.J.)

**Keywords:** thigmotropism, *Fusarium solani*, *Fusarium oxysporum*, scanning electron microscopy

## Abstract

Members of *Fusarium oxysporum* species complex (FOSC) and *Fusarium solani* species complex (FSSC) are common etiologic agents of opportunistic mycoses. Their hyphae grow via apical extension followed by invasion of host epithelial surfaces, particularly corneal and epithelial tissues. Thigmotropism is the contact-sensing response of these growing hyphae to change the direction of their apical growth in response to changes in the topography of their contacted surfaces, such as clefts between adjacent host cells. Investigations of fungal thigmotropism among etiologic agents of opportunistic mycoses are sparse. In this study, we assessed thigmotropic capacities of 10 fusarial strains. Thigmotropic activity was quantified using a chemotaxicell system followed by scanning electron microscopy (SEM). Isolates belonging to the FSSC had significantly greater rates of thigmotropism than FOSC strains (*p* < 0.0001). In summary, this work offers a creative method to assess thigmotropism, a crucial aspect of fungal pathogenesis, and, secondly, demonstrates potent thigmotropic capacities of fusarial isolates belonging to the FSSC.

## 1. Introduction

*Fusarium* species [1,2] are filamentous fungi commonly found in soil and frequently on plants and in plant debris [1,3,4,5,6,7]. Fusarial isolates, particularly members of the *Fusarium solani* species complex (FSSC) and the *Fusarium oxysporum* species complex (FOSC), are etiologic agents in both immunocompetent and immunosuppressed hosts of mycotic keratitis [8,9,10,11,12,13,14,15,16], fusariosis of the skin [12,17,18], and onychomycosis [1,18,19,20]. Additionally, these isolates are opportunistic pathogens in burn wound patients [21], following solid organ [22] and bone marrow transplantation [23,24], combat-related invasive fungal wound infections [25], central venous catheterization [26], and may disseminate into systemic fusariosis [27]. Mortality rates for disseminated fusariosis are severe, extending above 70% [28,29]. Among *Fusarium* spp., members of FSSC are the most virulent [1,9,26], recalcitrant and the most resistant to treatment [30,31,32]. Epidemiologically, environmental reservoirs and the colonization of a hospital water system with *Fusarium solani* and *Fusarium oxysporum* have been associated with opportunistic fusariosis [33,34]. In addition to impacting human and domestic animal health, fusarial species may impact agricultural yields [35,36,37,38], particularly reducing corn and soybean harvests in fields affected by fusariosis [39,40].

Thigmotropism is a distinctive fungal virulence factor enabling both opportunistic and primary filamentous fungus to detect a potential point of host tissue invasion by changing the direction of their growing hyphal apices as a response to alterations in the host tissue’s topography [41,42,43,44,45,46,47,48]. Thigmotropic ability facilitates hyphal invasion of host tissue, particularly in cleavages between host cells [41], between layers within nail plates in onychomycosis [49], and, additionally, along compromised host surfaces, such as abraded or injured corneal epithelium [14,15,16]. The focus of our study was to develop a method to compare thigmotropic abilities among fusarial isolates and to investigate thigmotropic capacities of isolates belonging to FSSC and FOSC, two major taxonomic groups within the diverse *Fusarium* genus [4,50,51,52,53,54] that are characterized contrastingly as weak and strong opportunists. In order to evaluate their thigmotropic capacities, we used scanning electron microscopy (SEM) to determine thigmotropic frequencies of each isolate’s hyphae that were in contact with isopore filters containing 3 µm diameter pores. There are a few reports documenting thigmotropism in the mycelial phase of *Candida albicans* [42,55,56,57,58,59], in addition to thigmotropic ability by *Aspergillus niger* [43] and malignant melanoma cells [60]. Our work detects differential thigmotropic capacities of fusarial opportunistic pathogens among animals.

The focus of our study was to examine thigmotropic capacities among FOSC and FSSC isolates and their comparative capacities. Although the capacity to produce proteases and toxins that damage host tissue, hydrophobicity of hyphae, and detoxification of host defensive compounds for filamentous fungi are described [1,61,62,63,64,65], little is known regarding thigmotropism among fusarial species [66]. Our work offers a method to quantify their thigmotropic capacity using SEM and, additionally, provides evidence of differential thigmotropic capacities among opportunistic members of the FOSC and FSSC.

## 2. Materials and Methods

Ten fusarial cultures including five *Fusarium oxysporum* species complex isolates and five *Fusarium solani* complex (5) isolates [67,68] (Table 1) were incubated on potato dextrose agar (PDA) slants at 35 °C in the dark for 2 days followed by 4 days at 25 °C to induce abundant sporulation.

**Figure 1:**
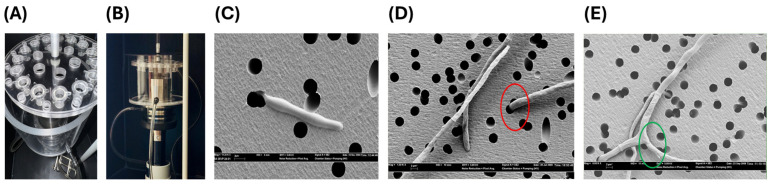
Sonication apparatus and thigmotropism assay materials. (**A**) Replicate chemotaxicells placed on customized sonication apparatus (**B**) for digitally controlled sonication of chemotaxicells containing fusarial suspensions post-incubation and prior to removal of individual filters from bottom of chemotaxicells for SEM analysis, (**C**) SEM of *F. solani* hypha thigmotroping through 3 µm pore, (**D**) SEM displaying a pore occupied by a hypha (red oval). Hyphae not occupying pores were measured as no thigmotropism. (**E**) Branching hyphae (green oval) not occupying pores were regarded as separate hyphae. Bars = 6 µm.

**Figure 2:**
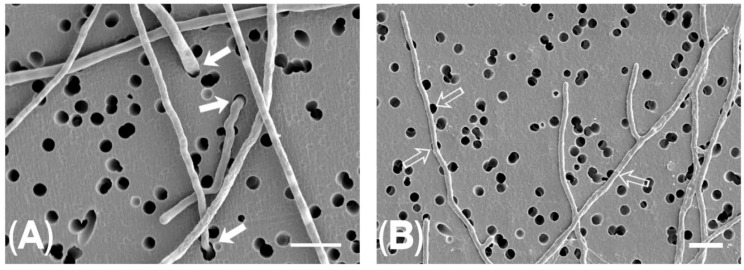
SEMs of fusarial hyphae grown within Chemotaxicells^®^. (**A**) SEM of exterior surface of Chemotaxicell^®^ isopore filter with thigmotroping hypha (TH) (solid arrows) of *F. solani* emerging from isopore pores 3 µm in diameter. Bar = 10 µm. (**B**) SEM with arrows (open arrows) pointing to non-thigmotroping hyphae (non-TH) of *F. oxysporum.* Bar = 10 µm. Bars are different lengths in images due to difference in magnification.

Each slant’s mycelial surface was covered with sterile reverse osmosis (RO) water and then gently scraped with a sterile glass Pasteur pipet to obtain a conidial suspension that was transferred to a sterile tube and vigorously vortexed. The resulting fungal suspensions were kept still for 45 min for hyphae to settle. Then, the homogenous conidia-containing layer was centrifuged at 350× *g* for 20 min, and the pellets were washed twice with sterile RO water [67]. Conidia were resuspended in RPMI-1640 medium at 1 × 10^5^ conidia/mL and incubated at 30 °C to induce germination [32]. RPMI stands for Roswell Park Memorial Institute and RPMI-1640 is a buffered modification of RPMI synthetic broth that is widely used for fungal and mammalian culture medium. The germinated suspensions were diluted to 1 × 10^4^ conidia/mL RPMI-1640 to prepare the fungal inoculum. Chemotaxicell^®^ inserts (pore size 3 µm; Kurabo, Osaka, Japan) were placed in wells of 24-well tissue culture plates. Then the chemotaxicell wells were filled with 700 µL of RPMI-1640, the inserts were inoculated with 500 µL of the germinated suspension, followed by incubation for 48 h at 27 °C to promote thigmotropism (Figure 1C). Next, the chemotaxicells were gently moved to a sonicator apparatus, digitally controlled, custom built Masonic sonicator (Model S-3000) (Misonix Incorporated, Farmingdale, NY, USA) holding 24 openings, each located 5 cm from the center of the 6.3 cm wide titanium alloy horn, allowing placement of chemotaxicells equidistance from the outer edge of the horn (Figure 1A,B). Following sonication, chemotaxicells were gently rinsed with sterile, RO water, fixed with 1% formaldehyde, treated with 2.5% glutaraldehyde in 0.05 M sodium cacodlyate buffer, dehydrated and critical point dried to prepare for SEM. Individual filters were removed, mounted outer surface up, sputter coated with 21 nm of gold and examined using a ZEISS SEM 35 VP (Carl Zeiss AG, Oberkochen, Baden-Württemberg, Germany) at 5 kV. The percent of pores occupied by thigmotroping hyphae in each filter membrane was determined by examining 63 views, each 57 µm × 33 µm, that were uniformly distributed within a 133 µm × 97 µm grid, divided into 50 rows and 37 columns. To quantify thigmotropism (Figure 1C,D,E, Figure 2) the number of hyphae occupying pores in each of the 63 view was determined and classified as thigmotropic responses, whereas hyphae not occupying pores were measured as non-thigmotropic. Percent thigmotropism = (number of occupying hyphae/total number of hyphae) × 100. Branching hyphae not occupying the pores were counted as separate hyphae. Supplementary Material (Table S1) is attached.

**Figure 3:**
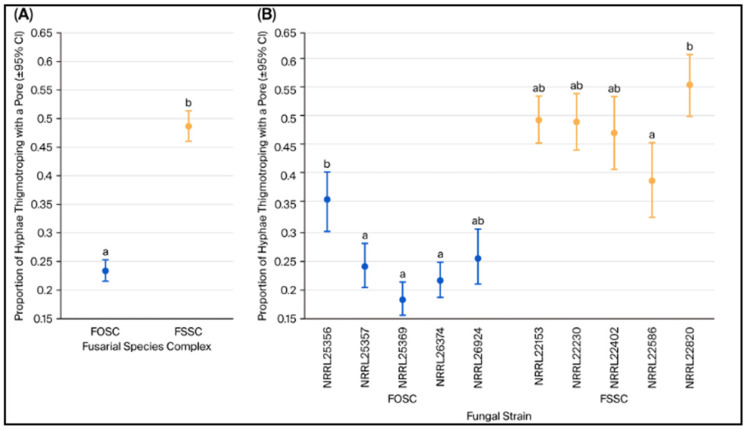
Comparison between FOSC and FSSC percent thigmotropism. (**A**) Comparison of thigmotroping proportions between FOSC and FSSC (five strains each). (**B**) Comparison of thigmotroping proportions among five FSSC and the five FOSC isolates on 3 µm pores of Chemotaxicell^®^ filters. Comparisons that are not significantly different are indicated with the same letter designation.

To compare the thigmotropic among 10 fusarial isolates, logistic regression analyses were performed using the thigmotropism rate as the proportional response and isolates as a 10-level classification variable (Figure 3). A main effects test comparing FOSC to FSSC, as well as within-complex strain comparisons, were performed using Sidak-adjusted statistical contrasts. All tests were performed at the 0.05 level of significance. The analysis was performed using R version 4.4.1.

## 3. Results and Discussion

The effect of species on the probability of thigmotropism was highly significant between fusarial isolates belonging to FSSC and FOSC (Odds ratio = 2.81, z = 14.634, *p* < 0.0001). Within the five strains of FSSC, the only significant difference was found between NRRL 22586 and NRRL 22820 (Odds ratio = 0.51, z = −3.805, Sidak-adjusted *p* = 0.0014). Among the five FOSC strains, significant differences were found between NRRL 25356 and NRRL 25369 (Odds ratio = 2.44, z = 5.918, Sidak-adjusted *p* < 0.0001) and between NRRL 25356 and NRRL 26374 (Odds ratio = 1.97, z = 4.661, Sidak-adjusted *p* < 0.0001). 

The present work developed a method to use a chemotaxicell system combined with SEM to quantitatively compare FOSC and FSSC thigmotropic capacities. Frequent turning of the FSSC hyphal apices into the 3 µm pores was apparent (Figure 2A) compared to less frequent thigmotroping by FOSC isolates (Figure 2B). In summary, greater thigmotropic capacity was found among fusaria belonging to the FSSC compared to fusaria belonging to the FOSC. The experiments demonstrated that the exterior surfaces of the isopore filters contained within Chemotaxicells® provide a means to elucidate fusarial thigmotropic capacities. Investigations into the ability of fungi to thigmotrope are sparse. The present study complements studies describing the role of chemotropism on directional hyphal growth responses in *Aspergillus niger* [43], modulation of thigmotropism in plants and animals by electric signaling [44,69], calcium-dependency of thigmotropism by *Candida* [45,46,70] *albicans*, and its attenuation by the calcium channel blocker verapamil [70], molecular mechanisms of mechanosensing and mechanosensitive ion channels in hyphal growth [45,71,72]. In summary, using a chemotaxicell system with SEM, we examined thigmotropic potentials of 10 fusarial strains, including five FSSC and five FOSC strains, demonstrating that the thigmotropic capacities of FSSC strains exceeded those demonstrated by FOSC strains. This study does not prove causation and is limited by the lack of a positive control. Experiments related to FSSC and FOSC isolates to explore adhesion to host epithelial or corneal cells, invasion of host tissues, penetration of epithelial barriers, virulence in ex vivo models, and correlation with clinical severity are needed to provide insights into comparative morbidity, mortality, and virulence. Moreover, experiments to analyze fusarial mechanisms of hyphal tip expansion and deformation during thigmotropism, fusarial thigmotropic responses to verapamil, an L-type calcium channel blocker that inhibits candidal hyphal thigmotropism without inhibiting hyphal elongation, and host-specific and tissue-specific variation between FSSC and FOSC thigmotropic expression may expand our understanding of the fusarial hyphal tip’s intriguing ability to thigmotrope [45,46,47,69,70,71,72,73].

## Figures and Tables

**Table 1:** Fusarial cultures examined in this study for thigmotropic capacities.

	Species/Haplotype	
*Fusarium oxysporum* species complex		
ARS *^a^* (NRRL *^b^* 25356)		CA *^c^* 92015
ARS (NRRL 25357)		CA 91142
ARS (NRRL 25369)		IMI *^d^* 312016
ARS (NRRL 25374)		IMI 190154
ARS (NRRL 26924)		
*Fusarium solani* species complex		
ARS (NRRL 22153)	FSSC 10-a	*^e^* ATCC 18099
ARS (NRRL 22230)	FSSC 17-b	ATCC 4934
ARS (NRRL 22402)	FSSC 23-a	*^f^* BBA 64954
ARS (NRRL 22586)	FSSC 13-b	BBA 64954
ARS (NRRL 22820)	*Fusarium virguliforme*	BBA 67586

*^a^* ARS is the United States Department of Agricultural Research Service. *^b^* NRRRL is the National Center for Agricultural Utilization Research and is the former name of the ARS. *^c^* CA is the Canadian Culture Collection *^d^* IMI is the International Mycological Institute, Egham, Surrey, England, now merged into the CABI (Centre for Agriculture and Bioscience International). *^e^* American Type Culture Collection, Manassas, VA. *^f^* Biologische Bundesanstalt für Land- und Forstwirtschaft, Institut für Mikrobiologie, Berlin, Germany.

## Data Availability

The original contributions presented in this study are included in the article/Supplementary Material. Further inquiries can be directed to the corresponding author.

## Supplementary Materials

The following supporting information can be downloaded at: https://www.mdpi.com/article/10.3390/jof12050344/s1, Table S1: SEM data for thigmotroping hyphae (TH) and non-thigmotroping hyphae (non-TH) FOSC and FSSC strains. Each replicate represents sixty-three views, each 57 µm × 33 µm, within 133 µm × 97 µm grids.
