# Supplementary material for: Differential Thigmotropic Capacities Among Fusarial Isolates: Fusarium solani Species Complex (FSSC) Isolates Most Potent
Source: J Fungi (Basel). 2026 May 7;12(5):344. doi: 10.3390/jof12050344 (PMC13207945; doi:10.3390/jof12050344)
Supplement: Supplementary file 1 [file jof-12-00344-s001.zip › jof-4211637-supplementary.pdf]

Table S1. SEM data for thigmotroping hyphae (TH) and non-thigmotroping hyphae (non-TH) FOSC and FSSC strains. Each replicate represents sixty-three views, each 57 μm x 33 μm, within 133 μm x 97 μm grids.

Fungus	Repli-cate	Total Number TH	Total Number Non-TH	% TH	Mean percentage TH (SD)
FOSC (NRRL 25356)	A	53	55	49.07	36.84 (11.46)
	B	44	123	26.35	
	C	20	37	35.09	
FOSC (NRRL 25357)	A	56	133	29.63	21.93 (8.39)
	B	51	169	23.18	
	C	10	67	12.99	
FOSC (NRRL 25369)	A	59	355	14.25	21.07 (10.85)
	B	46	91	33.58	
	C	24	132	15.38	
FOSC (NRRL 25374)	A	55	168	24.66	26.50 (3.65)
	B	38	179	17.51	
	C	65	226	22.34	
FOSC (NRRL 26924)	A	37	103	26.43	26.05 (3.19)
	B	27	92	22.69	
	C	18	44	29.03	
FSSC (NRRL 22153)	A	129	139	48.13	49.35 (4.34)
	B	70	83	45.75	
	C	91	77	54.17	
FSSC (NRRL 22230)	A	67	68	49.63	49.19 (9.57)
	B	72	51	58.54	
	C	54	83	39.42	
FSSC (NRRL 22402)	A	35	18	66.04	49.74 (14.35)
	B	39	61	39.00	
	C	38	48	44.19	
FSSC (NRRL 22586)	A	45	76	37.19	44.34 (12.19)
	B	30	50	37.50	
	C	7	5	58.33	
FSSC (NRRL 22820)	A	74	44	62.71	56.07 (9.07)

	B	59	70	45.74	
	C	49	33	59.76	
